# Tribological Performance of Additive Manufactured PLA-Based Parts

**DOI:** 10.3390/polym16172529

**Published:** 2024-09-06

**Authors:** Moises Batista, Irene Del Sol, Álvaro Gómez-Parra, Juan Manuel Vazquez-Martinez

**Affiliations:** Mechanical Engineering and Industrial Design Department, School of Engineering, University of Cadiz, Avda. de la Universidad de Cadiz, 10, E11519 Puerto Real, Spain; irene.delsol@uca.es (I.D.S.); alvaro.gomez@uca.es (Á.G.-P.)

**Keywords:** additive manufacturing, tribology, print parameters, wear resistance, advanced materials, PLA, polylactic acid

## Abstract

Polymer additive manufacturing has advanced from prototyping to producing essential parts with improved precision and versatility. Despite challenges like surface finish and wear resistance, new materials and metallic reinforcements in polymers have expanded its applications, enabling stronger, more durable parts for demanding industries like aerospace and structural engineering. This research investigates the tribological behaviour of FFF surfaces by integrating copper and aluminium reinforcement particles into a PLA (polylactic acid) matrix. Pin-on-disc tests were conducted to evaluate friction coefficients and wear rates. Statistical analysis was performed to study the correlation of the main process variables. The results confirmed that reinforced materials offer interesting characteristics despite their complex use, with the roughness of the fabricated parts increasing by more than 300%. This leads to an increase in the coefficient of friction, which is related to the variation in the material’s mechanical properties, as the hardness increases by more than 75% for materials reinforced with Al. Despite this, their performance is more stable, and the volume of material lost due to wear is reduced by half. These results highlight the potential of reinforced polymers to improve the performance and durability of components manufactured through additive processes.

## 1. Introduction

The emergence of additive manufacturing technologies has opened new frontiers in the design and production of components with optimised tribological properties. This advancement has revolutionised various fields of manufacturing engineering, enabling the creation of complex components with an unprecedented level of customisation. Additionally, it is a family of processes that allows for minimising the environmental impact of manufacturing processes.

Despite the potential of additive manufacturing, it is crucial to understand how the manufacturing process influences the final properties of parts. While there is considerable knowledge about certain properties, this is not the case for tribological properties. However, tribology is a key discipline in engineering, as it focuses on the study of friction and the wear of surfaces in relative motion, thereby centring on the behaviour of parts in service.

In this context, previous studies have shown that the mechanical properties of parts manufactured using additive processes can vary significantly depending on the manufacturing parameters, suggesting that their tribological behaviour could also be notably affected [1]. For instance, basic parameters such as layer orientation affect the friction and wear of manufactured parts. Vertically oriented parts tend to exhibit higher friction but lower wear depth compared to horizontally oriented ones [2]. This indicates that manufacturing orientation can influence the wear resistance and coefficient of friction of fabricated parts [3]. This demonstrates the complexity of achieving optimised tribological behaviour in parts made by additive manufacturing. This occurs in both metal and polymer additive manufacturing processes, although the latter are more widespread, despite the significant environmental impact of polymer use [4].

Among polymer additive manufacturing processes, fused filament fabrication (FFF) is one of the most popular due to its low cost, ease of use, and hybridisation [5]. In this process, also known as FDM (fused deposition modelling), manufacturing parameters control the mesostructure and, consequently, the behaviour of parts. This is important for their tribological behaviour as the structure of the parts affects their friction and wear [6]. Indeed, when comparing parts obtained through additive manufacturing with those produced by traditional methods such as moulding, additive manufacturing parts exhibit a higher coefficient of friction [7]. Therefore, optimising the structure and surface of the parts is necessary [8].

However, material selection also plays a crucial role. The choice of materials is critical to optimising the tribological properties of parts obtained through additive manufacturing [1]. In FFF, thermoplastic polymers in filament form are generally used, with ABS (acrylonitrile butadiene styrene) and PLA (polylactic acid) being the most common due to their ease of processing and mechanical properties [9,10]. ABS is a widely used material in additive manufacturing [11], but its low environmental performance does not recommend its use. PLA, on the other hand, is a biodegradable polymer considered a promising alternative to conventional petroleum-derived plastics due to its lower environmental impact, making its use highly interesting for optimised environmental performance [12,13].

However, the lack of standardisation of these materials has led to significant variability and complexity in their development. Simply adding a colour pigment can significantly affect the final properties of a material and even the tribological behaviour of parts [14,15]. In this context, where there is a wide variety of commercial filaments and materials play such a crucial role, little is known about the tribological behaviour of many materials. This is even more significant in the latest reinforced materials, where the aim is to exploit the special properties of the reinforcements.

For instance, the addition of silica nanoparticles to a PLA matrix has been shown to significantly reduce the coefficient of friction and wear rate [16]. A similar effect occurs with the addition of short carbon fibres, reducing the coefficient of friction and increasing wear resistance compared to pure PLA [17]. Conversely, the incorporation of graphite particles in an ABS matrix can increase the coefficient of friction, albeit at the expense of lower wear resistance [18]. This indicates that adding a reinforcement to a thermoplastic matrix allows for modulating the tribological properties of the material used in FFF.

To improve the environmental performance of the process, matrices with good environmental performance such as PLA should be used, along with reinforcements from industrial waste, such as metal particles. In cases where a thermoplastic material is reinforced with metal particles, it is expected that the addition of these particles will significantly improve certain mechanical properties, such as hardness, which will affect tribological performance and potentially improve it. However, it is important to remember that using particles from industrial waste that maintain or improve material properties represents a significant environmental contribution.

To achieve optimal tribological performance, metal particles with good tribological properties should be sought. Copper is known for its excellent tribological properties, including a low coefficient of friction that enhances performance in friction and wear applications. Specifically, copper has a coefficient of friction that can be advantageous for applications requiring low friction, such as bearings and other sliding parts [19]. Therefore, adding copper particles should improve the tribological performance of PLA parts manufactured using FFF.

It has been demonstrated that incorporating copper particles results in significantly better friction coefficients and wear resistance compared to pure PLA, suggesting that copper particles enhance the durability and efficiency of the material in wear applications [20]. However, there is limited knowledge on how these particles affect other important mechanical properties, such as hardness. There is also limited knowledge on other metal particles, such as aluminium, whose widespread industrial use makes it a great candidate for implementation.

On the other hand, incorporating these particles increases roughness and manufacturability issues [18,20,21]. Since the contact layers’ behaviour is critical in tribological performance, the air gap parameter is very important [3,22]. This is due to the influence of the roughness of the initial contact layers in creating a tribological pair. Therefore, given the semicylindrical geometry of deposited filaments in FDM, a part’s behaviour is very different from that of a continuous part made of the same material, complicating process modelling [23]. Hence, the knowledge of a specific tribological pair cannot be extrapolated to additively manufactured parts. Even the testing methods must be adapted, as observed by Gurrala et al. [24] in pin-on-disc tests, where normal load, sliding speed, and part orientation significantly influence the wear rate. This has been observed in other materials used in FFF [25]. Consequently, post-treatments are sometimes employed to improve wear resistance and control friction in components made by additive manufacturing [8]. However, this generates additional costs and reduces process efficiency, making it something to avoid.

Although significant advances have been made in understanding the tribological behaviour of parts obtained through additive manufacturing, challenges remain due to the variability in mechanical and tribological properties caused by the inherent anisotropy of printed parts, requiring further research. Reinforced filaments can improve tribological properties by incorporating particles that control these properties, but there is no existing knowledge about these filaments. Nor are there comparative studies that allow for understanding how these filaments behave. Thus, despite their potential to contribute to the environmental aspect of the process by using particles from industrial waste, their use is limited due to the lack of knowledge.

Therefore, this study aims to enhance the existing knowledge of these materials by comparing the tribological behaviour of components obtained using FFF techniques. To contribute to the environmental aspect of the process and improve the understanding of PLA as a biodegradable material, the influence of metallic reinforcement on two materials considered interesting will be studied—the first, copper, noted for its excellent tribological properties, and the second, aluminium, for its widespread use in various industrial applications. Thus, commercial filaments will be used under the same conditions to improve knowledge about the tribological behaviour of these materials and facilitate their industrial implementation, leading to the production of more efficient and durable components through additive manufacturing.

## 2. Materials and Methods

This article analyses the tribological behaviour of parts fabricated using FFF (fused filament fabrication), which, according to ISO/ASTM 52900:2021 [26], belongs to the material extrusion family, MEX (material extrusion). The objective of this research was to study the influence of a material (with and without reinforcement) on the tribological behaviour of parts manufactured using this process. To this end, commercially available PLA-based filament materials were selected, specifically, a general-purpose PLA filament, a high-performance PLA filament, a PLA filament reinforced with 20% aluminium particles, and a PLA filament reinforced with 20% copper particles. The geometries and morphologies of the particles were provided by the filament supplier. All selected filaments had a diameter of 1.75 mm and were supplied by Mas Toner D.I SLU. The selection of these reinforcements was made to compare a material of high industrial interest and wide consumption, such as aluminium, with a material of high tribological performance, such as copper. This was intended to contribute to the utilisation of industrial waste, thus favouring the environmental component of additive processes. These reinforced materials were compared with a general PLA filament and an advanced filament incorporating a series of additives, the composition of which is an industrial secret, that enhanced the mechanical properties of the materials. The general data regarding the mechanical properties of the materials studied, obtained from the supplier, are shown in Table 1.

**Figure 1:**
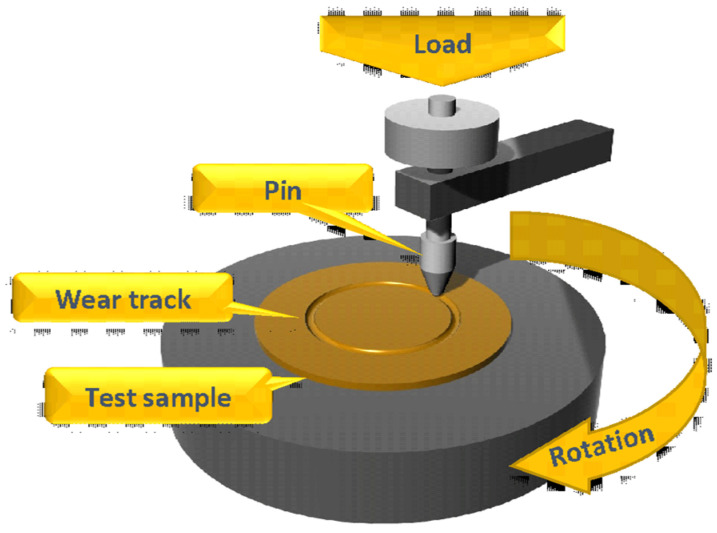
Pin-on-disc technique scheme.

For the tribological tests, circular specimens with a diameter of 70 mm and a thickness of 2 mm were fabricated using the FFF additive manufacturing process and tested using the pin-on-disc technique (Figure 1). In this test, a disc of one material is rotated against a pin of another material under a load to obtain their tribological interference.

For the fabrication of these specimens, FFF commercial equipment with a nozzle diameter of 0.4 mm was used. A series of parameters, such as an extrusion speed of 60 mm/s, an overlap of 55%, a build surface temperature set at 60 °C, and a 100% infill in the form of *Archimedean chords*, were fixed as constants. This pattern was selected with the intention that the test marks would align coherently with the deposition paths, avoiding seams that could affect tribological performance, as this has been shown to be a critical parameter. A series of variable parameters were also used. The parameters are listed in Table 2. The fabricated specimens were subjected to a pin-on-disc tribological test using Microtest series MT equipment (Microtest, Madrid, Spain). A stainless-steel pin in the form of a 3 mm diameter sphere of AISI 316L steel was used as the reference material. All tests were performed with a load of 15 N, a linear speed of 105 mm/s, and a length of 250 m. These parameters were selected based on previous studies [23,25]. Three repeated tests were performed on each sample, with a wear track radius of 10, 15, and 20 mm, while maintaining a constant linear speed. The tests followed the guidelines established by ASTM G99-17 [27]. However, since the standard preceded the impact of additive manufacturing and therefore does not take into account the specific characteristics of these processes, the tests were carried out taking into account previous studies on additive processes [23,25].

The coefficient of friction (CoF) data were analysed using marginal analyses, studying the evolution with sliding distance. Subsequently, an average CoF was calculated, discarding the first and last 50 m to account for stabilisation. Additionally, the average CoF for each material was determined as the mean of the three tests for each material. The amplitude of the CoF curves for each test was also calculated, expressed as the average difference between the three maximum values and the three minimum values.

Once tested, the surfaces of the specimens were characterised using stereoscopic optical microscopy with a Leica S9i (Leica, Wetzlar, Germany). Additionally, to characterise the groove thickness, a full characterisation of the specimens was performed using a variable-focus microscope, a Bruker Alicona G5+ (Bruker, Chicago, IL, USA, EEUU). A complete quadrant of each specimen was analysed, and the surface was measured before the test, as well as the depth and the width of the groove that appeared, to study the influence of the initial topographic roughness, Sa, Sz, and Sdc, with the depth and width of the groove. Three measurements for each parameter were made according to ISO 25178-2:2012 [28] and EUR 15178EN [29]. Sa is defined as the average height deviation of the surface from a mean line within the measurement area, Sz as the maximum amplitude of the surface, and Sdz provides a measure of how many peaks of a surface exceed a given height threshold per unit area.

In addition, in order to analyse the mechanical properties of the base materials, HMV 0.025 microhardness measurements were carried out for a time of 10 s with a Shimadzu HMV microhardness tester (Shimadzu, Kyoto, Japan).

All obtained data were subjected to statistical correlation and variance analysis.

## 3. Results

The results obtained from the different techniques used are analysed below.

### 3.1. Manufacturing Analysis

The manufacturing of FFF parts with PLA is well-studied and known due to its widespread use in this process. Tensile strength in the parallel direction to the layers can be significantly higher than in the perpendicular direction, indicating considerable anisotropy introduced during the printing process [30]. On the other hand, in the direction parallel to the layers, there is a high surface roughness due to the stair-stepping effect and the texture resulting from layer-by-layer deposition. This issue can be mitigated through post-printing treatments, which have been shown to significantly improve the surface quality of parts [15,31]. However, these treatments also alter the mechanical properties.

**Figure 2:**
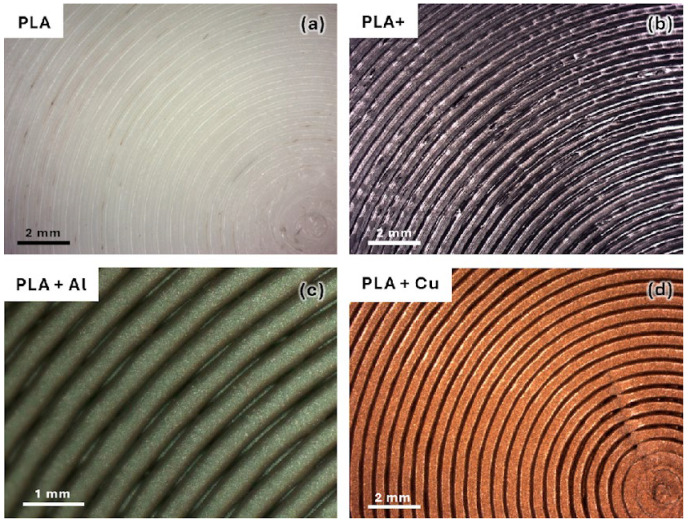
Manufactured specimen surfaces. (**a**) Using PLA filaments. (**b**) Using high-performance PLA filaments. (**c**) Using PLA filaments reinforced with 20% Al particles. (**d**) Using PLA filaments reinforced with 20% Cu particles.

In the perpendicular direction to the layers, roughness depends greatly on the manufacturing parameters and material composition. Figure 2 shows images of the specimens fabricated with different materials.

As can be seen, the incorporation of any additive modifies the extrusion behaviour. When this additive is a reinforcement, partial nozzle occlusions are generated, causing the deposited filaments to be unstable, which is clearly seen in the specimens with metallic reinforcements. This causes larger gaps in the fabricated parts.

These defects negatively affect mechanical properties, such as tensile strength and stiffness, and may originate from suboptimal printing parameters or inconsistencies in filament extrusion [32].

**Figure 3:**
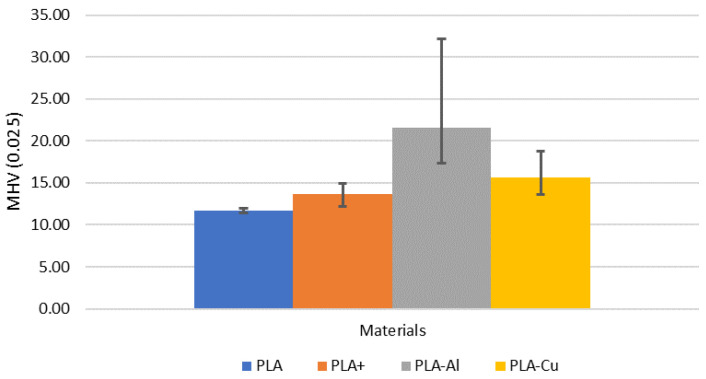
Microhardness of the materials studied.

In order to analyse the properties of each material, an analysis of the microhardness of the material was carried out (Figure 3). As can be seen, the incorporation of reinforcement hardens the material, as expected, and is directly related to the hardness of the reinforcement incorporated. On the other hand, it can be observed how the lack of homogeneity in the distribution of the reinforcement causes a greater dispersion of the data. This will be very important in the accumulation of the reinforcement and its possible hardening by compression of the material.

### 3.2. Surface Quality

**Figure 4:**
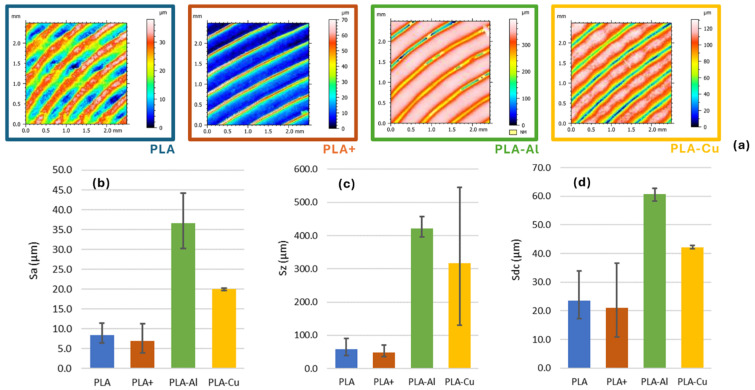
(**a**) Topological representations using FMV techniques. (**b**) Comparative surface quality of different test specimens obtained in Sa terms. (**c**) Comparative surface quality of different test specimens obtained in Sz terms. (**d**) Comparative surface quality of different test specimens obtained in Sdc terms.

The most evident manifestation of these defects is the change in the surface quality of the parts. This causes irregularities on the surfaces of the parts. Figure 4 shows the quality measures obtained for the parts fabricated with different materials.

Moreover, surface defects and roughness must have a clear significance for the tribological behaviour of PLA parts, especially those obtained by additive manufacturing. Surface roughness directly influences the friction and wear of printed parts, affecting their performance in applications where contact and relative motion between surfaces are critical.

However, it should be noted that the characteristic roughness of the FFF process is due to the stair-stepping effect of layer addition, which increases with layer thickness and can raise the coefficient of friction between contact surfaces. In this case, an *Archimedean chord* infill strategy can be selected, and the pin-on-disc (PoD) test will encounter this geometry instead of the stair-stepped layer. This approach was chosen because it represents the contact surface between parts rather than the lateral surface. Thus, the movement of the print head induces the analysed roughness, and potential surface irregularities act as additional contact points, increasing the sliding resistance.

Furthermore, voids known as air gaps can form between the deposited filaments, which are inherent to parts printed via FFF. This effect plays a crucial role in tribological behaviour, as these voids not only weaken the internal structure of the parts but can also trap wear particles, acting as additional abrasives that accelerate the wear of contact surfaces. Proper management of printing parameters, such as extrusion temperature and deposition speed, can minimise these defects and enhance wear resistance [33].

Finally, the anisotropy in the mechanical properties of printed parts also affects their tribological behaviour. Differences in strength and stiffness between printed layers can cause uneven wear and greater susceptibility to fracture under tribological loads. Print orientation and infill pattern are key factors that must be optimised to improve the homogeneity and durability of PLA parts [32].

### 3.3. Friction Analysis

**Figure 5:**
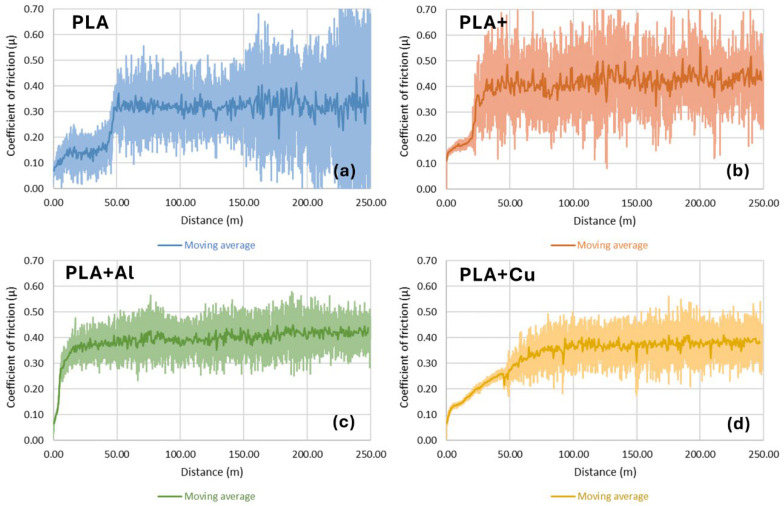
Evolution of the coefficient of friction for the different materials studied with a 10 mm of test radius. (**a**) Using PLA filaments. (**b**) Using high-performance PLA filaments. (**c**) Using PLA filaments reinforced with 20% Al particles. (**d**) Using PLA filaments reinforced with 20% Cu particles.

The reinforcements added to the material seemed to have a greater importance than the defects they generated. As shown in Figure 5, the reinforcements seemed to stabilise the material’s behaviour, minimising the data-dispersion effect.

**Figure 6:**
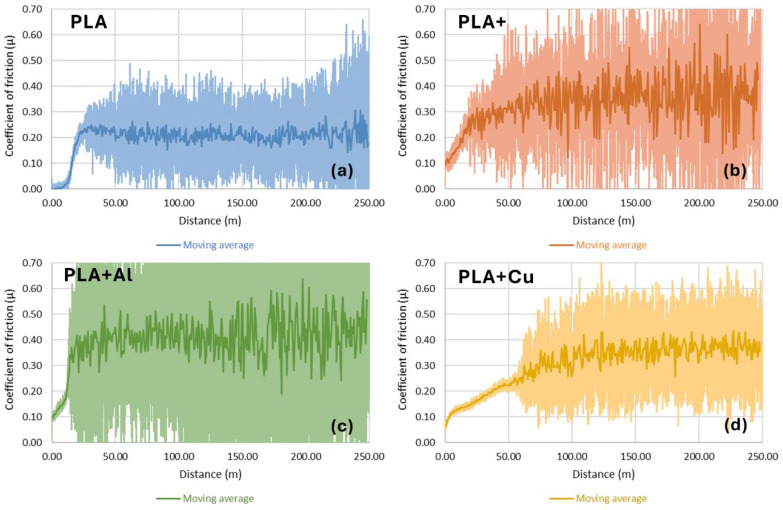
Evolution of the coefficient of friction for the different materials studied with a 20 mm of test radius. (**a**) Using PLA filaments. (**b**) Using high-performance PLA filaments. (**c**) Using PLA filaments reinforced with 20% Al particles. (**d**) Using PLA filaments reinforced with 20% Cu particles.

However, this did not occur when the radius increased, despite maintaining a constant linear speed (Figure 6). This may have been conditioned by the stick–slip phenomenon. This phenomenon occurs when sliding movements alternate between sticking and slipping phases, generating vibrations and noise. This behaviour is particularly problematic in applications where smooth and controlled movement is required. Studies have shown that surface roughness and lack of homogeneity can exacerbate this effect, increasing friction and wear [34].

Thus, an amplitude increase manifests a smaller stick–slip cycle. This may occur due to the sliding trajectory, as at a smaller radius the pin slides more frequently over the same path, causing it to pass over the same area in a shorter time. This causes greater material compression and sliding, potentially filling the voids and porosities that appear in FDM parts more quickly, resulting in a more stable groove and, therefore, greater stability. This concurs with studies where a lower void density generated more stable behaviour of parts [33].

Moreover, reinforcement can significantly increase hardness and other mechanical properties. This is one of the most interesting properties of these composites. This is because the metallic particles restrict the movement of polymer chains, improving scratch and deformation resistance. A study found that adding copper and aluminium powder to an unsaturated polyester resin significantly increased the composite material’s hardness, its maximum value being reached with a 15% volume fraction of metallic powder [35].

This hardening can favour the formation of a stable groove and stabilise a polymer’s behaviour.

**Figure 7:**
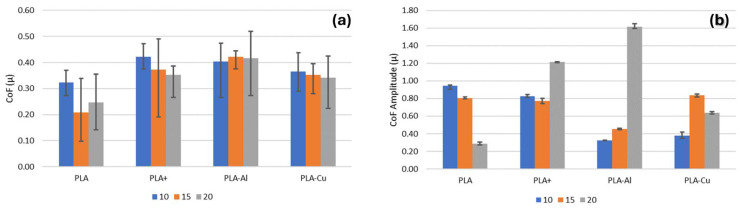
Evolution of average friction coefficient (**a**) and amplitude (**b**).

Therefore, despite the apparent dependence of the coefficient of friction on roughness, as previously mentioned, this trend was not observed in the average values of the coefficient of friction (Figure 7). Thus, the average coefficient of friction is relatively stable, regardless of the material or test radius (Figure 7a). This could be influenced by surface hardening, which may promote the formation of a groove and minimise the negative effect of roughness in reinforced polymers, resulting in a compensation of wear effects and, consequently, no significantly different average values being generated.

**Figure 8:**
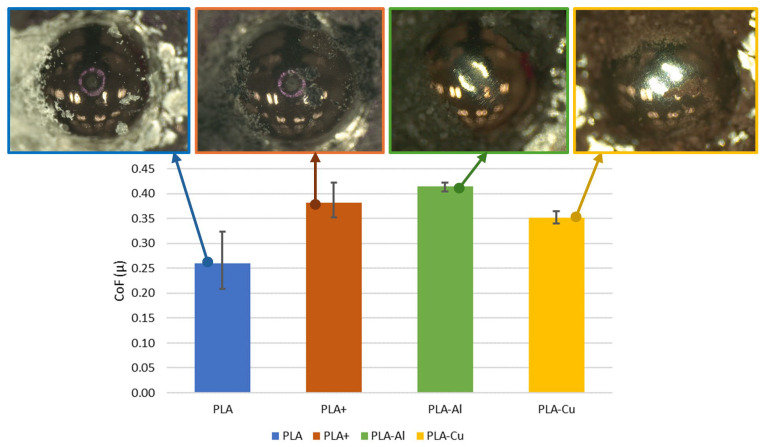
Comparison of the average friction coefficient for each material and its influence on the pin.

**Figure 9:**
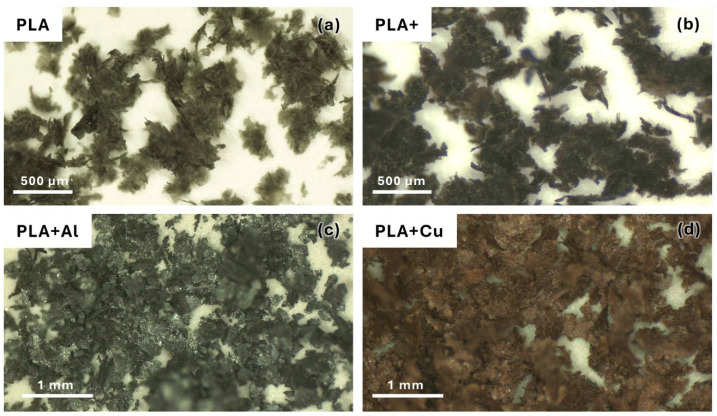
Comparison of the morphology of the detached material (debris) with a 15 mm of test radius. (**a**) Using PLA filaments. (**b**) Using high-performance PLA filaments. (**c**) Using PLA filaments reinforced with 20% Al particles. (**d**) Using PLA filaments reinforced with 20% Cu particles.

However, there was a noticeable dependence on material hardness when comparing hardness with the average coefficient of friction per material, as shown in Figure 8. Harder materials exhibit slightly higher average coefficients of friction. Additionally, this hardness caused more damage to the pin in the form of scratches (Figure 8). It can also be observed that some of the detached particles were projected and trapped on the pin. This deposition was not permanent, as these particles were not adhered and detached, with no adhesion phenomena observed on the pin. On the other hand, the detached particles (debris) showed a very similar morphology (Figure 9), although the shine of the metal particles could be seen. Therefore, it can be concluded that not all particles remained in the base material to harden it, resulting in partial hardening. Regarding the coefficient of friction, the incorporation of a reinforcement or additive does not seem to improve this parameter. Similarly, on the one hand, the elliptical geometry of the surface promoted friction, as has been observed in other studies, where geometries contrary to sliding can result in higher friction coefficients, up to 0.3 in the case of PLA [25]. On the other hand, it is important to note that, in this study, the manufacturing parameters were kept constant, though they are a determining factor that can modulate friction behaviour. Poor manufacturing parameters can worsen tribological performance [23]. However, it is worth highlighting that the worst data obtained without optimisation were similar to the friction coefficient data obtained for PETG without optimisation, which is a material that exhibits good tribological performance [23].

**Figure 10:**
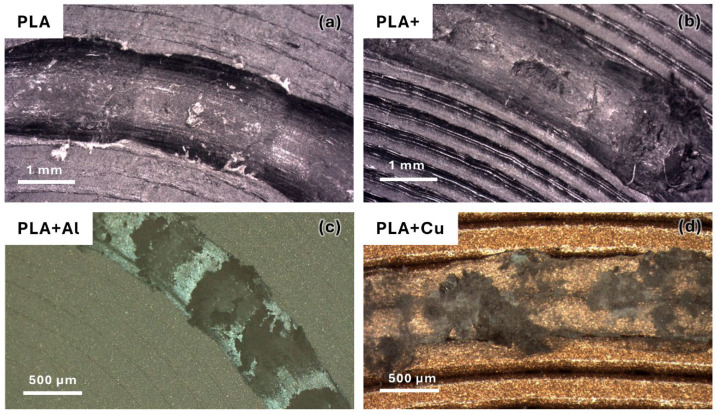
Comparison of the morphology of the grooves with a 10 mm of test radius. (**a**) Using PLA filaments. (**b**) Using high-performance PLA filaments. (**c**) Using PLA filaments reinforced with 20% Al particles. (**d**) Using PLA filaments reinforced with 20% Cu particles.

When analysing the morphology of the grooves (Figure 10), a direct relationship between the hardness of the material and the groove morphology can be observed, which is consistent with previous observations. As the hardness increased, material extrusion phenomena appeared on the groove, with the most notable ones occurring in the material reinforced with Al, which had the highest hardness. Additionally, the morphology of this extruded material is very characteristic in the case of reinforced materials, similar to the behaviour of detached particles, suggesting that they may behave similarly. Thus, in materials without particle reinforcement, a more stable groove appears, which contrasts with what can be observed in Figure 2. Therefore, it can be inferred that this material accumulation caused by the previously mentioned stick–slip phenomenon does not affect the stability of the friction process. On the other hand, this accumulated material did not adhere to the pin, so there was hardly any abrasive or adhesive behaviour on the pin, as has been previously observed. Only the friction and sliding phenomenon characteristic of this type of part produced by FFF was present.

**Figure 11:**
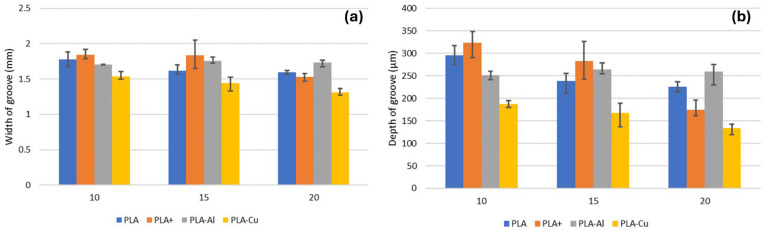
(**a**) Evolution of the width of the groove wear. (**b**) Evolution of the maximum depth of the groove wear.

When analysing the geometry of the grooves, regarding the width and depth of the wear grooves (Figure 11), it can be observed that there is no clear trend with the type of material, and the trend appears to be the opposite of that observed in the coefficient of friction, where the PLA reinforced with copper exhibits the highest coefficient of friction but offers narrower and shallower grooves. Additionally, the potential hardening effect caused by the incorporation of the reinforcement does not seem to play a significant role, as no clear trend can be observed. It is also noted that, in this case, the incorporation of the reinforcement appears to improve wear resistance. For the unreinforced PLA material, the enhancement of mechanical properties achieved in the advanced PLA results in poorer performance, likely caused by the increased hardness and toughness of the material.

**Figure 12:**
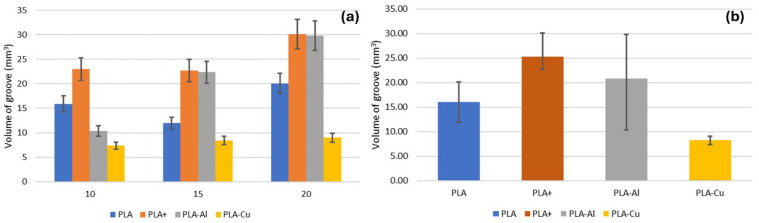
(**a**) Evolution of the volume of the groove wear. (**b**) Volume of the groove wear in relation to the function of the material.

This wear track geometry will result in a loss of material volume. As observed in previous analyses, there does not seem to be a clear trend with the test radius (Figure 12a). Although there appears to be a slight trend towards growth, this would be due to the wear effect on the different test radii. With smaller radii, the number of passes of the pin over the same area of the sample increases, which corresponds to an increase in the number of wear cycles. Consequently, larger radii result in shallower groove depths and narrower groove widths. However, since the affected area is larger, the groove volume is greater. Therefore, analysing groove width and depth provides more information than volume, despite the latter being the factor recommended by current standards.

On the other hand, as observed in previous cases, the best wear performance was exhibited by the material reinforced with copper, regardless of the test radius (Figure 12b). The other materials did not seem to exhibit improved performance, although the incorporation of aluminium does not appear to have a significantly negative effect. This does not align with previous studies [20]. However, wear analysis is a complex discipline influenced by many factors, and additive manufacturing parts affected by different parameters can produce distinct effects. This indicates that systematic tests must be conducted to determine which factors control behaviour in order to optimise the performance of these materials.

### 3.4. Statistical Analysis

To understand how wear characteristics vary between different materials, ANOVA tests were performed on the test data. Statistically significant tests (*p* < 0.05) indicated substantial differences in key variable means between the materials. The results are detailed below.

#### 3.4.1. Surface Roughness (Sa)

When comparing all materials, there was a clearly significant difference regarding surface roughness (F-statistic = 31.41, *p*-value = 0.000089). This indicates a significant difference in average surface roughness (Sa) among the different materials. This suggests that some materials exhibit rougher surfaces than others, which should influence tribological behaviour.

On the other hand, comparing materials with and without reinforcement revealed a significant difference in surface roughness (F-statistic = 20.65, *p*-value = 0.0011). The reinforced materials exhibited higher surface roughness compared to the non-reinforced materials. This suggests that reinforcements increase surface irregularity, possibly due to greater wear resistance, leading to a rougher surface.

There was a significant difference in surface roughness (Sa) between the studied materials (F-statistic = 20.65, *p*-value = 0.0011). Reinforced materials had a higher surface roughness compared to the non-reinforced materials. This suggests that reinforcements increase surface irregularity, possibly due to higher wear resistance, resulting in a rougher surface.

This concurs with the general behaviour of composite materials, since reinforcement incorporation tends to increase the hardness and wear resistance of materials, leading to generally rougher surfaces [36].

When applied to the case of FDM, where reinforced materials show increased load-bearing capacity of FDM-fabricated parts with carbon fibre-reinforced nylon, this can contribute to higher surface roughness due to increased wear resistance [37].

#### 3.4.2. Maximum Roughness (Sz)

Regarding maximum roughness (Sz), the material had significant importance (F-statistic = 9.03, *p*-value = 0.006). The maximum roughness (Sz) measures showed significant differences between the materials, indicating variations in the maximum irregularities of the worn surfaces.

Reinforcement also showed significant differences (F-statistic = 27.31, *p*-value = 0.000). Reinforced materials exhibited higher maximum roughness values, indicating that these materials’ surfaces had more pronounced irregularity peaks after wear tests. This concurs with previous studies by Singh et al. [38], who observed that the presence of reinforcements in materials increased irregularity peaks. This same behaviour has been observed in other cases. For example, in functional prototypes based on Al_2_O_3_-reinforced nylon6 produced via FDM, it was observed that reinforcement-particle use can lead to higher wear resistance and affect tribological behaviour [39].

#### 3.4.3. Surface Profile (Sdc)

Regarding the surface profile (Sdc), significant differences were observed between the studied materials (F-statistic = 14.95, *p*-value = 0.0012), suggesting that some materials have surfaces with more pronounced profiles after wear.

When analysing the influence of reinforcement, a significant difference was observed between the reinforced and non-reinforced materials (F-statistic = 23.54, *p*-value = 0.001). The reinforced materials had higher surface profiles, indicating greater resistance to deformation under wear conditions.

The incorporation of reinforcement particles in 3D-printed materials can enhance their mechanical resistance and modify their surface profiles. This concurs with previous studies by Eutionnat-Diffo et al. [40], which demonstrated that the properties of the material and printing parameters significantly influence the wear resistance and surface profile of conductive PLA filaments printed on textiles.

This is because reinforced materials typically have higher surface profiles due to their increased rigidity and resistance to deformation [41].

#### 3.4.4. Average Friction Coefficient

In the case of the average coefficient of friction, when comparing the studied materials, the average coefficient of friction (average CoF) varied significantly (F-statistic = 10.84, *p*-value = 0.0034), indicating that the resistance to sliding was slightly higher in the reinforced materials.

Although differences were found between reinforced and non-reinforced materials, these differences were not statistically significant (F-statistic = 3.06, *p*-value = 0.111). This could be because the addition of reinforcements does not substantially alter the surface interaction with wear elements in terms of average friction or due to the presence of secondary behaviours that minimise these differences, as previously mentioned.

However, as observed in previous studies, reinforcement influence on the friction coefficient can vary depending on the reinforcement nature and wear type, but it is not always significant [42].

#### 3.4.5. Friction Coefficient Amplitude

Regarding the amplitude of the coefficient of friction, no significant differences were observed among the studied materials (F-statistic = 0.49, *p*-value = 0.701), suggesting that the variability in the coefficient of friction is similar across the different materials. This implies that other mechanisms, such as the occurrence of stick–slip phenomena, are more responsible for this variability.

Similarly, no significant differences were observed in the amplitude of the coefficient of friction between the reinforced and non-reinforced materials (F-statistic = 0.03, *p*-value = 0.859). This suggests that friction coefficient variability is similar regardless of the presence of reinforcements. In a study on graphene integration in polylactic acid (PLA) via 3D printing, it was found that while graphene reinforcement improved wear resistance, there was no significant change in friction coefficient variability, supporting the observation that reinforcements may not affect friction coefficient amplitude [43].

Therefore, it appears that friction coefficient amplitude may depend more on test conditions than material type and on the previously discussed friction mechanisms, concurring with previous studies [44].

#### 3.4.6. Wear Groove Width

No significant differences were found in the average groove width among the studied materials (F-statistic = 1.09, *p*-value = 0.407), indicating that the wear width does not vary substantially with the material.

Additionally, the average groove width did not show significant differences between the reinforced and non-reinforced materials (F-statistic = 1.18, *p*-value = 0.304). This indicates that the reinforcement did not significantly affect the width of the produced wear.

The optimisation study of additive manufacturing parameters by Portoacă et al. [45] indicates that factors such as infill percentage and layer thickness have a greater influence on wear groove width than reinforcement addition, supporting the observation that reinforcements may not have a significant impact on this aspect.

Wear groove width may be more influenced by applied forces and test duration than material composition [46].

#### 3.4.7. Maximum Wear Groove Depth

No significant differences were found in the maximum groove depth among the studied materials (F-statistic = 3.33, *p*-value = 0.077), although the *p*-value suggests that this factor might be influenced by the material.

Similarly, there does not appear to have been a significant effect from the use of reinforcements (F-statistic = 2.15, *p*-value = 0.173). This suggests that the presence of reinforcements does not notably influence the depth of the wear.

In the study by Gadelmoula and Aldahash [47] on friction and wear resistance of carbon fibre-reinforced polyamide 12, it was found that while reinforcements improved wear resistance, groove depth did not show significant variations, consistent with the observation that reinforcements may not affect wear depth.

Wear groove depth may be similar between reinforced and non-reinforced materials due to factors such as load distribution and inherent material resistance [48].

#### 3.4.8. Volume of the Groove Wear

Although the dimensional values of the groove did not seem to be significantly influenced by the material, there did appear to be a significant difference regarding the worn volume (F-statistic = 4.86, *p*-value = 0.033). This difference may be defined by this parameter being more comprehensive than simple dimensional values.

However, this dependence did not seem to be influenced by the reinforcement (F-statistic = 1.77, *p*-value = 0.214). This means that the volume of material worn is largely conditioned by the nature of the material itself, which is evident. However, it is not necessarily affected by the incorporation of a specific reinforcement but rather by the inherent properties of the material, which is consistent with previous results [20].

#### 3.4.9. Summary of Statistical Analysis

The analysis shows that reinforced materials have a higher surface roughness and maximum roughness, as well as a more pronounced surface profile compared to non-reinforced materials. However, no significant differences were observed in mean friction coefficient, friction coefficient amplitude, groove width, or maximum groove depth between the reinforced and non-reinforced materials.

This indicates that reinforcement has a notable impact on surface roughness and profile characteristics but does not significantly affect measures related to friction coefficient and wear groove dimensions.

Furthermore, no significant differences were observed in friction coefficient amplitude, groove width, or maximum groove depth between different materials. These results indicate that while surface characteristics and friction vary, wear groove dimensions are more consistent among the evaluated materials.

Studying the correlation between different parameters reveals the following:

- Sa and Sz: These parameters have a strong positive correlation (0.97), indicating that as average surface roughness (Sa) increases, maximum surface roughness (Sz) also tends to increase.
- CoF mean and CoF amplitude: The mean friction coefficient and its amplitude are moderately correlated (0.59), suggesting that variations in the mean friction coefficient are somewhat related to changes in its amplitude.
- Material impact: To understand the impact of different materials, we need to examine how these correlations differ when segmented by material type. This would require deeper analysis using grouped data.
- Volume of the groove wear and maximum wear groove depth: There is a strong positive correlation between the volume of the groove wear and the maximum wear groove depth. This indicates that as the maximum average depth of the groove increases, the volume of the groove also increases significantly.

**Figure 13:**
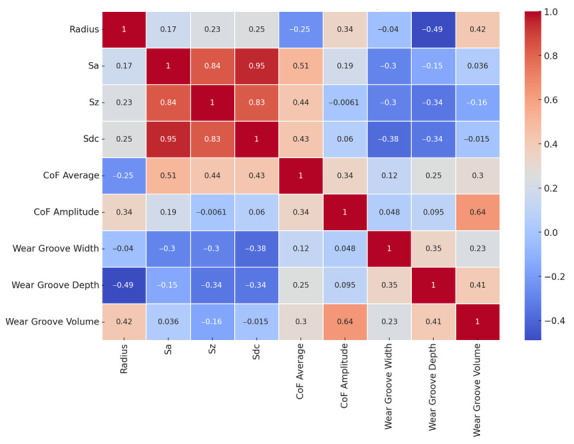
Correlation matrix of wear test data.

The Figure 13 shows a correlation matrix for the study variables:

## 4. Conclusions

Additive manufacturing, especially that based on fused filament fabrication (FFF), enables the creation of complex geometries and advanced materials beyond conventional methods. Improved understanding of their tribological properties can greatly expand their applications.

Selecting materials and adding reinforcements, like metal particles, improve wear resistance in FFF-fabricated parts without necessarily enhancing the friction coefficient. This approach is also environmentally beneficial, as using industrial waste materials can match or exceed the performance of non-reinforced alternatives.

During fabrication, reinforced materials behave differently from non-reinforced ones, likely due to partial nozzle occlusion. This reinforcement also impacts surface quality, resulting in increased surface roughness.

Reinforced materials show more stable behaviour and less data variability, but increased surface roughness and heterogeneity can worsen stick–slip effects, negatively impacting tribological performance. Reinforced materials have higher surface irregularity, suggesting improved wear resistance but with potential drawbacks in terms of tribological properties. Post-wear analysis reveals more pronounced irregularity peaks, indicating greater deformation resistance and possible enhanced durability in demanding applications.

Although behavioural differences between materials were observed, they were not statistically significant, indicating that adding reinforcements does not substantially affect surface interaction or mean friction. Similarly, there were no significant differences in friction coefficient variability, wear track width, or depth, suggesting that reinforcements do not significantly impact these factors.

In summary, the results obtained showed increases in the hardness of the PLA starting material of up to 83% for aluminium and 33% for copper. In the case of surface finish, higher roughness values were confirmed for metallic reinforced materials compared to PLA and advanced PLA. Moreover, a reduction in the coefficient of friction and less dispersion of the values obtained for lower running radii were observed, implying a significantly more uniform sliding. This fact resulted in reductions of approximately 55% in the worn volume of the copper-particle-reinforced material when compared to the initial non-reinforced PLA material and of 71% when compared to PLA+.

## Figures and Tables

**Table 1:** Material properties.

	Specific Gravity (g/cm^3^)	Impact Strength (KJ/m^2^)	Tensile Strength (MPa)	Tensile Modulus (Mpa)	Elongation at Break (%)	Softening Temperature (°C)
PLA	1.2–1.4	6.5–7.0	55–60	3.2–3.5	5.5–6.0	50–55
PLA + Advanced	1.2–1.4	7.0–7.5	50–53	3.5–3.8	5.8–6.2	55–60
PLA + Al (20%)	1.4–1.6	4.0–4.5	43–48	4.7–4.9	4.8–5.2	52–55
PLA + Cu (20%)	2.4–2.6	4.0–4.5	38–42	4.0–4.3	4.3–4.6	50–55

**Table 2:** Materials and manufacturing parameters.

Material	T (°C)	Layer Thickness (mm)	Extrusion Velocity (mm/s)	Overlap	Bed Temperature (°C)	Infill
PLA	210	0.25	60	55%	60	Archimedean chords (100%)
PLA + Advanced						
PLA + Al (20%)						
PLA + Cu (20%)						

## Data Availability

The original contributions presented in the study are included in the article; further inquiries can be directed to the corresponding authors.
